# Study on the daily dose and serum concentration of clozapine in psychiatric patients and possible influencing factors of serum concentration

**DOI:** 10.1186/s12888-023-05078-z

**Published:** 2023-08-15

**Authors:** Taixiu Liu, Peng Gao, Chuange Xie, Heng Zhang, Zheng Shi, Ruirui Chen

**Affiliations:** Department of Clinical Laboratory, Shandong Daizhuang Hospital, Jining, 272051 China

**Keywords:** Clozapine, Serum concentration, Daily dose, Therapeutic drug monitoring (TDM)

## Abstract

**Background:**

Clozapine is the most effective drug for treatment-resistant schizophrenia, and the dosage and concentration of clozapine in the treatment of mental illness vary greatly in different populations and are affected by many factors.

**Methods:**

The serum clozapine concentration of 3734 psychiatric patients was detected, and data on daily dose, sex, age and other medical records were collected for statistical analysis.

**Results:**

The mean daily dose, mean serum concentration and mean C/D (concentration/dose) ratio of clozapine were 191.02 ± 113.47 mg/day, 326.15 ± 235.66 ng/mL and 1.94 ± 1.25 ng/mL per mg/day, respectively. There was difference in daily dose between sexes, and females had higher daily dose (*p* <0.01), higher serum clozapine concentrations (*p* < 0.01) and higher C/D ratios (*p* < 0.01). There were significant differences in daily dose (*p* < 0.001), serum drug concentration (*p* < 0.001) and C/D ratio (*p* < 0.001) among different age groups. The daily dose decreased with age (p for trend < 0.001), and the C/D ratio increased with age (p for trend < 0.001). Inpatients and outpatients had no difference in daily dose, but inpatients had higher serum concentration (*p* < 0.001) and C/D ratio (*p* < 0.001). There was no difference in daily dose among different occupations, but there were significant differences in serum concentration (*p* < 0.001) and C/D ratio (*p* < 0.001), and unemployed patients may have higher serum concentration and C/D ratio. Duration of disease, comorbidity, marital status, and psychotic type may influence the daily dose and serum concentration.

**Conclusions:**

The effective daily dose and serum concentration of clozapine in the study area may be lower than recommended levels, and women have higher serum concentrations and slower metabolic rates. With increasing age, the daily dose decreases, and the metabolic rate slows. Inpatient status and occupation of patients may influence the serum concentration and metabolic rate of clozapine.

## Background

Clozapine is a second-generation antipsychotic that mainly blocks serotonin (5-HT2A) and dopamine (DA1) receptors in the brain to treat psychosis. In addition, clozapine can directly inhibit the upwards activation system of the brain stem reticular structure and has a relatively powerful sedative and hypnotic effect. Clinically, clozapine is mainly used for treatment-resistant schizophrenia or treatment-resistant bipolar disorder; it can improve the positive symptoms of psychosis to a certain extent and have an effect on the negative symptoms [1]. Clozapine can be used for acute and chronic schizophrenia and has a certain therapeutic effect on fantasies, delusions and hallucinations. It is also useful for treating emotional symptoms such as depression, guilt, and anxiety, as well as mania and other psychiatric disorders [2]. Furthermore, clozapine is the only FDA-approved drug to prevent suicidal behaviour [3].

Major side effects of clozapine include strong sedative effects, more anticholinergic effects, common dizziness, fatigue, drowsiness, sweating, salivation, nausea, vomiting, dry mouth, tachycardia, constipation and postural hypotension. Second, the more common symptoms are increased appetite and weight gain. Third, it can also cause elevated blood sugar and lipids [4]. Fourth, the serious adverse effects are agranulocytosis and secondary infection. Clozapine is currently recognized as a fairly effective drug in the treatment of schizophrenia and represents the most effective pharmacotherapy for treatment-resistant psychosis [5]. In addition, clozapine was associated with a reduction in overall mortality compared with other second-generation antipsychotics [6]. Clozapine has these unique effects that are not matched by other antipsychotic drugs, such as risperidone and olanzapine [7–9]. Because clozapine reduces granulocytes, a serious side effect, it is not considered the first choice of antipsychotic drug at present.

Therapeutic drug monitoring (TDM) quantifies and interprets the concentration of the drug in plasma or serum and examines the correlation between the dose of the drug and the concentration of the drug in the blood for each patient to yield the highest therapeutic effect and the lowest risk of adverse drug reactions/toxicity [10]. Based on empirical evidence, clozapine is a strongly recommended drug for TDM (Level 1) [11]. To ensure the efficacy of clozapine and reduce its side effects, blood clozapine concentrations should be monitored during clozapine therapy [12]. In this study, the relationship between clozapine dosage and serum drug concentration and the factors that may affect serum drug concentration were studied from the perspective of a population in eastern China to provide a basis and reference for the clinical application of clozapine.

## Methods

### Samples

All samples in this study were collected from patients in Shandong Daizhuang Hospital. The included subjects were diagnosed with schizophrenia (paranoid schizophrenia, undifferentiated schizophrenia and residual schizophrenia), affective (mood) disorder (bipolar disorder, recurrent depressive disorder, manic and major depressive episode) and others (organic, including symptomatic, mental disorders, dissociation [conversion] disorder, behavioural and emotional disorders that usually begin in childhood and adolescence, mental retardation, generalized anxiety disorder, obsessive compulsive disorder, specific personality disorder) by at least 2 psychiatrists according to the *International Classification of Diseases, 10th Revision (ICD-10)* or *Diagnostic and Statistical Manual of Mental Disorders* (*DSM–5*). The inclusion criteria were as follows: (1) patients with mental disorders diagnosed in our hospital, (2) patients with available medical records, and (3) patients taking clozapine. None of the subjects had obvious signs of clozapine poisoning or significant adverse drug reactions. The patients were instructed to take clozapine orally starting with a small dose; the initial dose was 25 mg/time, 2–3 times a day, and gradually increased to the conventional therapeutic dose according to the instructions.

In a steady state, drug intake equals drug elimination over a period of time, and the steady state valley concentration (Cmin) is usually quantitative. The Cmin of most drugs in the steady state (fixed dose treatment with at least 4–6 half-lives) has been used as a standard procedure and is recommended in therapeutic drug monitoring (TDM). The elimination half-life of clozapine is approximately 12–16 h on average [11], and serum drug concentrations were measured after 4 to 6 metabolic half-lives of fixed doses of clozapine. Patients took the drug at approximately 9–11 pm, and blood was drawn from the patient at approximately 6–8 am the next day. According to the clinical treatment requirements, the psychiatrist gave the patient a medical order for clozapine blood concentration monitoring, and the nurse drew 5 ml of fasting venous blood with a vacuum vessel without other components and immediately sent it for examination.

### Detection of serum clozapine concentration

The clozapine serum concentration was measured using high-performance liquid chromatography (HPLC) at the Clinical Drug Concentration Monitoring Laboratory, Department of Clinical Laboratory, Shandong Daizhuang Hospital. The received blood samples were placed in a temperature box at 37 °C for several minutes to accelerate solidification and centrifuged at 4000 r/min for 10 min after solidification.

One millilitre of the supernatant was added to a 10-ml glass tube, and 20 µl of the internal standard solution and 100 µL NaOH (concentration of 0.1 mol/L) were added and then shaken for several seconds. Then, 5 ml n-pentane was added and shaken for 1 min. The supernatant was transferred to another 10-ml glass tube, dried in a water bath at 70 °C, removed, cooled to room temperature and re-dissolved in 100 µl of mobile phase. After shaking for a few seconds, the supernatant was centrifuged at 2000 r/min for 5 min, and 30 µl of the supernatant was taken in the intubation and sent to the machine for analysis.

HPLC chromatographic conditions were as follows: mobile phase (methanol: water: tetramethylethylenediamine: acetic acid = 677:330:2.2:1.76), flow rate: 0.8 mL/min, column: SB-C18, detection wavelength: 254 nm, column temperature: 40℃, injection volume: 20 µl, injection time: 12 min. Internal standard solution: 2-amino-5-chloro-2-fluorophenone (5 µg/mL).

### C/D ratio and medical record information

The results of clozapine serum concentration were looked up in the Laboratory Information System (LIS). The daily dose of clozapine for psychiatric patients was obtained by consulting the doctor’s orders in the medical records. The serum concentration/daily dose ratio (C/D ratio) was obtained by calculating the ratio of serum clozapine concentration (ng/mL) to daily dose (mg/day), which represented the metabolism and clearance rate of clozapine in vivo [13]. C/D ratios are inversely related to total clearance; the lower the C/D ratio is, the faster the clearance rate of clozapine in vivo; in contrast, the higher the C/D ratio is, the smaller the clearance rate of clozapine in vivo [14].

No names were collected in the case inquiry or medical advice in order to protect patient privacy, and the medical record number was used as the inquiry identifier. The medical records of each subject were collected and analysed, including gender, age, ethnicity, marital status, mental illness type, residence, medication amount, etc.

### Statistical analysis

Data with a normal distribution will be described by the mean ± SD, and data with a non-normal distribution will be described by the median (interquartile range). Spearman correlation was used for correlation analysis; the Mann‒Whitney test and Kruskal‒Wallis test were used to compare the data between groups. In addition, univariate and multivariate linear regression analyses and trend tests were also used to analyse the data. Values of *p* < 0.05 were considered statistically significant. Analysis was computed using SPSS Statistics version 22.0 (IBM Corporation, Armonk, NY, USA).

## Results

### Demographic characteristics

A total of 3734 subjects were included in this study. All medical records were available and ranged in age from 10 to 75 years old, with an average age of 38.18 ± 11.75 years old. A total of 2424 male patients and 1310 female patients were included. The duration of the diseases ranged from 1 to 600 months, with an average of 195.63 ± 118.56 months. In this study, the psychiatric disorders treated with clozapine were mainly schizophrenia (75.92%) and mood disorder (22.25%). Other psychiatric disorders treated with clozapine, including “organic, including symptomatic, mental disorders” and “behavioural and emotional disorders that usually begin in childhood and adolescence”, accounted for 1.83% of the included subjects. Paranoid schizophrenia, undifferentiated schizophrenia, and bipolar disorder were the most commonly treated disorders with clozapine (Table 1).

**Table 1:** Types of psychiatric disorders treated with clozapine (*n* = 3734)

Classification of psychiatric disorders	Number	Percentage (%)
Schizophrenia (*n* = 2835)		
Paranoid schizophrenia	1425	38.16
Undifferentiated schizophrenia,	1403	37.57
Residual schizophrenia	7	0.19
Affective (mood) disorder(*n* = 831)		
Bipolar Disorder	779	20.86
Recurrent depressive disorder	24	0.64
Manic	19	0.51
Major depressive episode	9	0.24
Others (*n* = 68)		
Organic, including symptomatic, mental disorders	30	0.80
Dissociation [conversion] disorder	26	0.70
Behavioural and emotional disorders that usually begin in childhood and adolescence	4	0.11
Mental retardation	4	0.11
Generalized Anxiety Disorder	2	0.05
Obsessive compulsive disorder	1	0.03
Specific personality disorder	1	0.03

### Results of daily dose, serum concentration and C/D ratio

Clozapine was prescribed once daily to three times daily by the psychiatrist, depending on the patient’s condition. The daily doses ranged from 12.5 mg/day to 825 mg/day, and the average daily dose was 191.02 ± 113.47 mg/day. The serum concentrations of clozapine ranged from 27.44 ng/mL to 1884.23 ng/mL, with an average of 326.15 ± 235.66 ng/mL. The C/D ratio ranged from 0.18 to 12.84, with an average of 1.94 ± 1.25. The Spearman correlation analysis showed a positive correlation between daily dose and serum concentration (*r* = 0.594, *p* < 0.01) (Table 2).

**Table 2:** Clozapine dose, serum concentration and the C/D ratio

Variables	Mean ± SD	Median (interquartile range)	*r*
Daily dose (mg/day)	191.02 ± 113.47	175.00(100.00–250.00)	0.594^**a^
Serum concentration (ng/mL)	326.15 ± 235.66	272.36(148.09–445.98)	
C/D ratio	1.94 ± 1.25	1.67(1.10–2.47)	

^a^Spearman correlation coefficient between daily dose and serum concentration

^**^p < 0.01

**Fig. 1:**
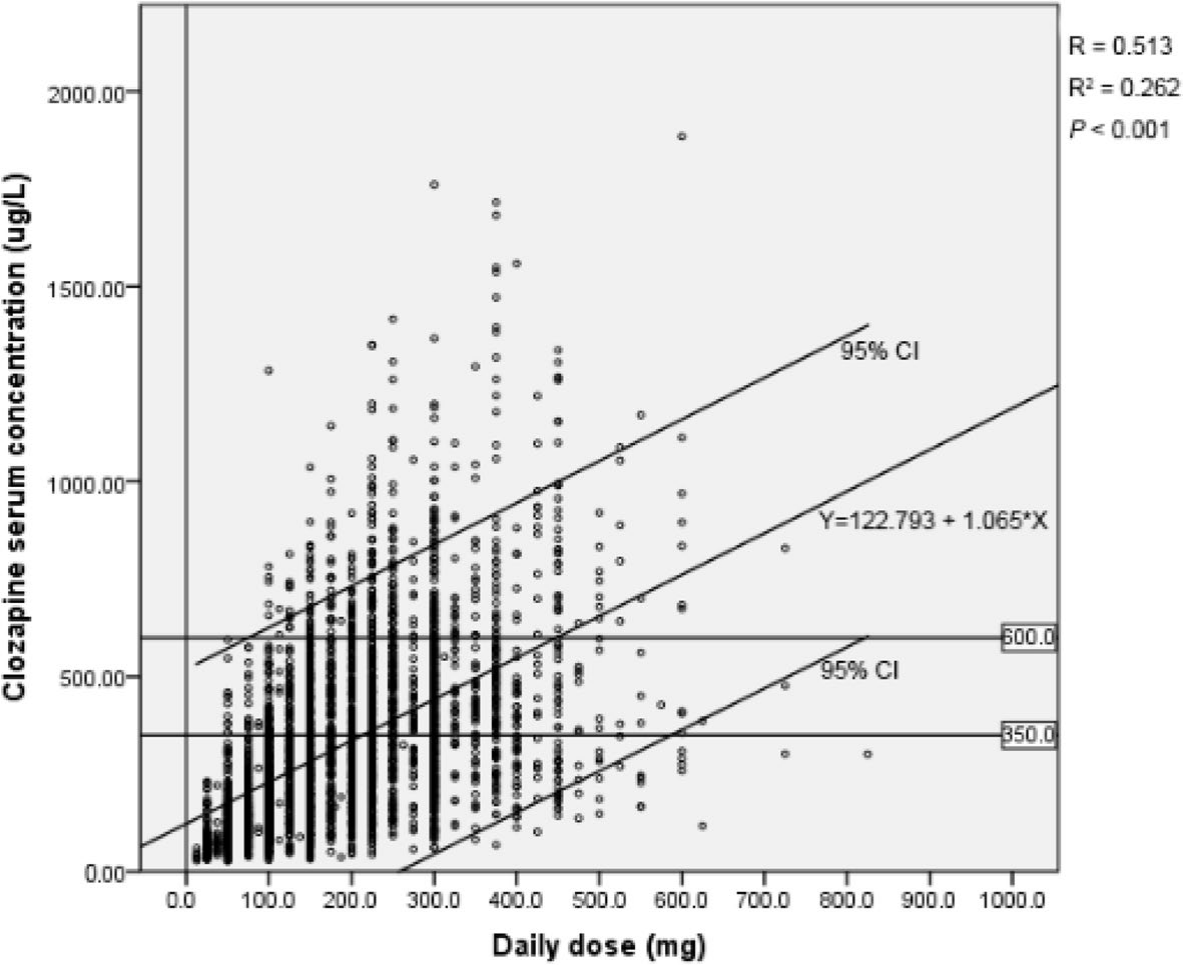
Relationship between the daily dose of clozapine and serum concentration. The linear regression equation of clozapine daily dose and concentration was Y = 122.793 + 1.065*x, *R* = 0.513, *p* < 0.001

Taking the clozapine daily dose as the independent variable and the serum drug concentration as the dependent variable, regression analysis showed that the regression coefficient was b = 1.067 (*R* = 0.513, *p* < 0.001) (Fig. 1).

The subjects were divided into four groups according to the daily dose: <100 mg/day group, 100–200 mg/day group, 201–400 mg/day group and > 400 mg/day group. The daily dose of clozapine in psychiatric patients between 100 and 400 mg/day accounted for 77.93% of the subjects, and the daily doses of 50–350 mg/d accounted for 87.50%. Through trend test analysis, the clozapine serum concentration showed an increasing trend with increasing drug dose (*p* < 0.001), and the C/D ratio showed a decreasing trend with increasing daily drug dose (*p* < 0.001) (Table 3).

**Table 3:** Trend test of different dose groups and different serum concentration groups

			Daily dose (mg/day)			Serum concentration (ng/mL)			C/D Ratio	
Groups	n	Mean ± SD	Median (interquartile range)	*p*	Mean ± SD	Median (interquartile range)	*p*	Mean ± SD	Median (interquartile range)	*p*
Daily dose (mg/day)										
< 100	663	53.28 ± 18.12	50.00(50.00–75.00)		131.38 ± 90.74	104.72(70.83–161.15)		2.57 ± 1.64	2.08(1.54–3.07)	
100–200	1666	145.37 ± 35.01	150.00(100.00–175.00)	0.000	289.09 ± 175.81	252.99(155.09–379.81)	0.000	2.03 ± 1.21	1.75(1.13–2.69)	0.000
201–400	1244	287.30 ± 52.27	300.00(250.00–300.00)		448.54 ± 250.42	406.93(270.53–567.25)		1.60 ± 0.91	1.39(0.97–2.03)	
> 400	161	486.65 ± 68.58	450.00(450.00–500.00)		566.00 ± 326.99	501.03(283.86–805.97)		1.19 ± 0.70	1.07(0.59–1.66)	
Serum concentration (ug/L)										
< 350	2344	153.91 ± 101.16	150.00(75.00–200.00)		183.33 ± 86.97	174.10(110.25–255.72)		1.56 ± 1.02	1.34(0.87–1.95)	
350–600	1048	239.59 ± 99.17	225.00(150.00–300.00)	0.000	471.79 ± 82.37	462.06(400.09–535.34)	0.000	2.37 ± 1.25	2.05(1.52–2.89)	0.000
> 600	342	296.53 ± 112.01	300.00(225.00–375.00)		858.70 ± 212.87	796.19(702.64–920.71)		3.28 ± 1.36	3.04(2.23–3.96)	

The subjects were divided into three groups according to the serum concentration: <350 ng/mL, 350–600 ng/mL and > 600 ng/mL. The serum clozapine concentration in most patients was < 350 ng/mL, accounting for 62.77%. Only 28.07% of the patients had clozapine concentrations between 350 and 600 ng/mL. A total of 9.16% of patients had clozapine concentrations > 600 ng/mL, among which 62 patients (1.66%) had clozapine concentrations > 1000 ng/mL and 1 patient had clozapine concentrations > 1800 ng/mL. The concentration range of 50–600 ng/mL accounted for 86.20% of the population in this study. Through trend test analysis, the C/D ratio showed an increasing trend with increasing serum drug concentration (*p* < 0.001) (Table 3).

### Comparison of clozapine daily dose, serum concentration and C/D ratios among different groups

Subjects were grouped according to different attributes. According to age, the psychiatric patients were divided into four groups: the 10-17-year-old group, 18-44-year-old group, 45-59-year-old group and 60-75-year-old group. More than half of the subjects were in the 18–44 age range. According to the duration of the disease, the patients were divided into two groups: the < 180 months group and the ≥ 180 months group. According to marital status, the patients were divided into three groups: single, married and divorced. According to occupation, the patients were divided into four groups: unemployed, farmers, workers and others. The patients were divided into two groups according to whether the diseases were associated with somatic diseases: the psychiatric disorder group and the psychiatric disorder combined with somatic disease (comorbidities) group. Additional background information is presented in Table 4.

**Table 4:** Baseline characteristics of the included patients (*N* = 3734)

Variables	n	Percentage (%)
Sex		
Female	1310	35.08
Male	2424	64.92
Age group(year)		
10–17	116	3.11
18–44	2418	64.76
45–59	1089	29.16
60–75	111	2.97
Duration (month)		
< 180	1924	51.53
≥ 180	1810	48.47
Ethnicity		
Han	3692	98.88
Hui	42	1.12
Marital status		
Single	1435	38.43
Married	1992	53.35
Divorced	307	8.22
Occupation		
unemployed	1718	46.01
Farmer	1091	29.22
Worker	468	12.53
Others	457	12.24
Comorbidity or not		
psychiatric disorders	3424	91.70
Comorbidities	310	8.30
Psychotic type		
Schizophrenia	2835	75.92
Bipolar disorder	779	20.86
Other types	120	3.21
Inpatients/outpatients		
Inpatients	3209	85.94
Outpatients	525	14.06
Province		
Shandong	3674	98.39
Others	60	1.61

Through statistical analysis, there was significant difference in the daily dose of clozapine between males and females (*p* < 0.01), there was a significant difference in the serum concentration of clozapine between males and females, and the serum concentration in females was higher (*p* < 0.01). There was also a statistically significant difference in the C/D ratio between males and females, and the C/D ratio in females was higher (*p* < 0.01).

There were significant differences in the daily dose of clozapine in different age groups, and the daily dose in the 10–17 age group was the highest (*p* < 0.01). Correspondingly, there were significant differences in serum concentrations among different age groups. There were significant differences in the C/D ratio among different age groups, and the C/D ratio in the 10–17 age group was the lowest (*p* < 0.01). There were significant differences in the daily dose, serum concentration and C/D ratio between the two groups with different disease durations. Compared with the > 180-month duration group, the daily dose in the ≤ 180-month duration group was higher (*p* < 0.01), and the serum concentration and C/D ratio in the < 180-month duration group were lower (*p* < 0.01).

Psychiatric disorders treated with clozapine were mainly schizophrenia and bipolar disorder, with few other diseases. There were significant differences in daily dose, serum concentration and C/D ratio among schizophrenia, bipolar disorder and other types of mental diseases. The schizophrenia group had the highest daily dose (p < 0.01) and serum concentration (*p* < 0.01). There was no significant difference in daily dose between outpatients and inpatients, but there was a significant difference in serum concentration (*p* < 0.01) and C/D ratio (*p* < 0.01). The serum concentration and C/D ratio in inpatients were higher.

There were significant differences in the daily dose serum concentration and C/D ratio between the psychiatric disorder group and the comorbidity group. The daily dose in the psychiatric disorder group was higher than that in the comorbidities group (*p* < 0.01), and the serum concentration and C/D ratio in the psychiatric disorder group were lower than those in the comorbidities group (*p* < 0.05 and *p* < 0.01, respectively). There were significant differences among different occupations in serum concentration (*p* < 0.01) and C/D ratio (*p* < 0.01), and the C/D ratio in the unemployed group was the highest (Table 5).

**Table 5:** Comparison of clozapine dose, serum concentration and C/D ratio among different variable groups

		Daily dose (mg)			Serum concentration (ng/mL)			C/D Ratios		
Variables	n	Mean ± SD	Median (interquartile range)	*P*	Mean ± SD	Median (interquartile range)	*P*	Mean ± SD	Median (interquartile range)	*P*
Gender										
Female	1310	194.77 ± 107.31	175.00 (100.00–250.00)	0.009^a^	373.37 ± 248.72	320.66 (179.30–508.36)	0.000^a^	2.17 ± 1.34	1.88 (1.25–2.72)	0.000^a^
Male	2424	188.99 ± 116.63	150.00 (100.00–250.00)		300.63 ± 224.24	249.30 (131.06–403.78)		1.83 ± 1.19	1.55 (1.02–2.32)	
Age group (year)										
10–17	116	216.27 ± 125.77	200.00 (100.00–300.00)	0.000^b^	338.65 ± 265.38	308.03 (113.66–490.33)	0.000^b^	1.61 ± 0.92	1.36 (0.86–1.98)	0.000^b^
18–44	2418	193.00 ± 115.91	175.00 (100.00–275.00)		307.94 ± 233.20	249.40 (137.74–410.38)		1.81 ± 1.14	1.56 (1.02–2.29)	
45–59	1089	188.65 ± 108.78	150.00 (100.00–250.00)		361.99 ± 236.25	329.30 (174.70–489.60)		2.20 ± 1.42	1.90 (1.27–2.73)	
60–75	111	144.59 ± 71.78	125.00 (100.00–200.00)		358.25 ± 207.07	343.13 (185.77–472.19)		2.67 ± 1.56	2.27 (1.55–3.38)	
Duration (month)										
≤ 180	1924	204.29 ± 116.87	200.00 (100.00–300.00)	0.000^a^	318.31 ± 239.00	259.64 (142.60–427.60)	0.003^a^	1.75 ± 1.11	1.47 (1.01–2.18)	0.000^a^
> 180	1810	176.91 ± 107.99	150.00 (100.00–225.00)		334.48 ± 231.82	285.00 (152.64–458.75)		2.15 ± 1.36	1.87 (1.24–2.73)	
Inpatients/outpatients										
Inpatients	3209	188.85 ± 110.24	150.00 (100.00–250.00)	0.097^a^	343.72 ± 239.35	294.66 (161.98–465.91)	0.000^a^	2.06 ± 1.27	1.78 (1.19–2.59)	0.000^a^
Outpatients	525	204.29 ± 130.79	200.00 (100.00–300.00)		218.75 ± 177.34	172.72 (95.02–279.92)		1.25 ± 0.83	1.05 (0.65–1.62)	
Psychiatric classification										
Schizophrenia	2835	196.23 ± 116.14	175.00 (100.00–275.00)	0.000^b^	339.02 ± 241.59	284.83 (157.56–459.65)	0.000^b^	1.98 ± 1.29	1.70 (1.11–2.51)	0.034^b^
Bipolar disorder	779	176.29 ± 105.53	150.00 (100.00–225.00)		288.80 ± 209.00	234.49 (129.68–388.50)		1.82 ± 1.10	1.59 (1.08–2.29)	
Other types	120	163.54 ± 111.34	150.00 (50.00–225.00)		264.62 ± 222.83	199.87 (109.82–331.52)		1.94 ± 1.31	1.71 (0.99–2.37)	
Comorbidity or not										
Psychiatric disorders	3424	193.16 ± 112.57	175.00 (100.00–250.00)	0.000^a^	325.84 ± 239.20	267.06 (145.14–447.17)	0.044^a^	1.89 ± 1.19	1.63 (1.07–2.40)	0.000^a^
Comorbidities	310	167.34 ± 120.58	150.00 (100.00–200.00)		329.60 ± 192.58	316.44 (179.28–434.09)		2.51 ± 1.69	2.16 (1.44–3.28)	
Marital status										
Single	1435	199.61 ± 119.93	175.00 (100.00–300.00)		311.30 ± 222.63	258.11 (138.40–429.40)		1.82 ± 1.23	1.54 (1.01–2.29)	
married	1992	186.31 ± 109.68	150.00 (100.00–250.00)	0.015^b^	335.07 ± 239.53	281.97 (152.88–450.50)	0.027^b^	2.00 ± 1.19	1.77 (1.15–2.57)	0.000^b^
divorced	307	181.39 ± 103.90	150.00 (100.00–225.00)		337.68 ± 265.36	275.50 (150.02–465.99)		2.17 ± 1.64	1.78 (1.12–2.70)	
Occupation										
unemployed	1718	189.26 ± 106.65	175.00 (100.00–250.00)	0.426^b^	336.94 ± 228.71	291.61 (161.67–462.02)	0.000^b^	2.02 ± 1.25	1.74 (1.17–2.58)	0.000^b^
Farmer	1091	188.10 ± 106.42	175.00 (100.00–250.00)		321.17 ± 244.27	252.07 (140.51–432.51)		1.88 ± 1.20	1.62 (1.05–2.39)	
Worker	468	191.53 ± 131.32	150.00 (100.00–250.00)		278.84 ± 184.37	253.39 (129.83–390.40)		1.88 ± 1.43	1.53 (1.00–2.41)	
Others	457	204.05 ± 132.79	175.00 (100.00–300.00)		345.94 ± 277.56	268.62 (128.74–483.43)		1.87 ± 1.17	1.63 (1.04–2.31)	

^a^Mann‒Whitney test

^b^Kruskal‒Wallis test

### Trend tests of dose, concentration, and C/D ratio between age groups

**Fig. 2:**
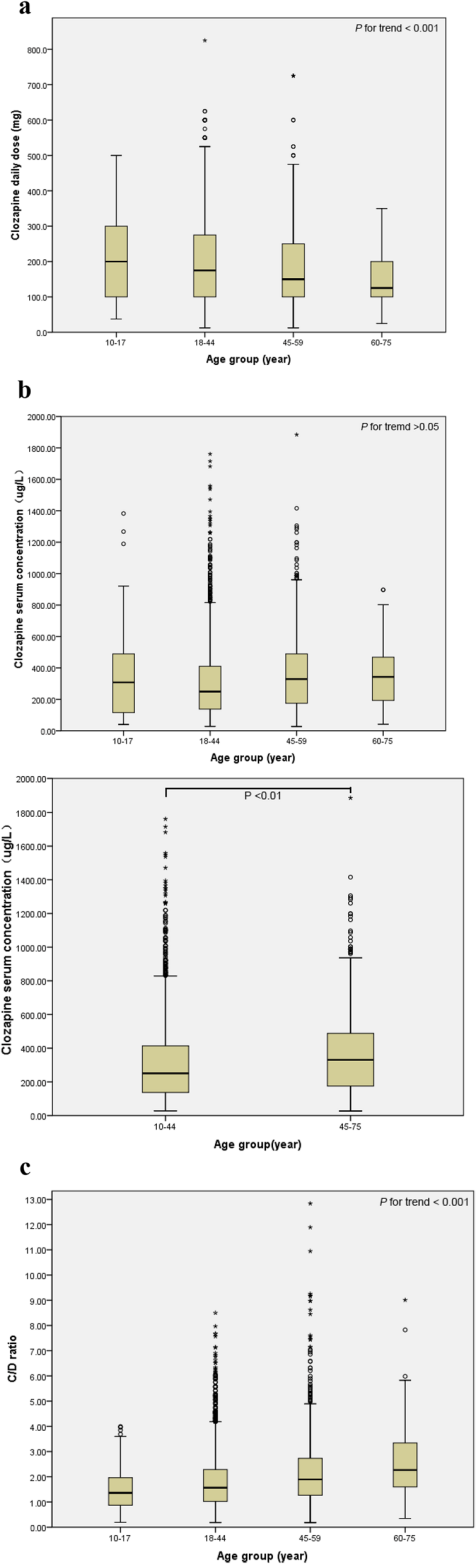
**a** Relationship between daily dose of clozapine and age groups. With increasing age, the daily dose of clozapine tended to decrease. **b1** Relationship between clozapine serum concentrations and age groups. Although there were significant differences in the serum concentrations of clozapine among age groups, there was no significant trend in the daily dose of clozapine with age. **b2** Relationship between clozapine serum concentration and age groups.When the subjects were divided into two age groups, 10-44 years old and 45-75 years old, the serum concentration of clozapine was significantly different between the two groups (*P* <0.01), and the serum concentration of clozapine in the 45-75 years old group was higher than that in the 10-44 years old group. **c** Relationship between the C/D ratio and age group. With increasing age, the C/D ratio tended to increase. With increasing age, the daily dose of clozapine tended to decrease

 The statistical results of this study showed that there were significant differences in the daily dose, serum concentration and C/D ratio among different age groups of psychiatric patients. According to trend analysis results, the daily dose of clozapine in psychiatric patients decreased with age (*p* < 0.001) (Fig. 2a). Serum concentration in the four age groups did not show an increasing trend with age (Fig. 2b1), but when the subjects were divided into 10–44 and 45–75 age groups according to their ages, there was a significant difference in serum drug concentration between the 10–44 and 45–75 age groups (*p* < 0.01), and the serum concentration in the 10–44 age group was lower (Fig. 2b2). The C/D ratio increased with age (*p* < 0.001) (Fig. 2c).

### Regression model and identifying factors

We used a regression model to further examine the factors that influenced serum clozapine concentration. After univariate and multivariate regression analyses revealed that daily dose, sex, age, inpatients/outpatients, and occupation (worker) were significant factors (*p* = 0.000). The duration of the disease and the type of mental illness were significant factors in the univariate regression model (*p* < 0.05) but were not significant in the multivariate regression model (*p* > 0.05). Thus, daily dose, sex, age and occupation are factors that predict serum clozapine concentration (Table 6).

**Table 6:** Predictors associated with clozapine serum concentration in the linear regression model

Variables		Univariate model		Multivariate model	
		B (95%CI)	*p*	B (95%CI)	*p*
Daily dose (mg/day)		1.065 (1.007;1.122)	0.000	1.091 (1.036;1.146)	0.000
Sex	Male	-72.738 (-88.411;-57.065)	0.00	-71.282 (-84.858;-57.706)	0.000
Age (year)		1.716 (1.075;2.357)	0.000	2.250 (1.488; 3.011)	0.000
Inpatients/outpatients	Outpatients	-124.971 (-146.352;-103.589)	0.000	-129.023 (-147.713;-110.333)	0.000
Duration (month)	≥ 180	16.170 (1.048;31.292)	0.036	-8.611 (-24.952; 7.730)	0.302
Comorbidity or not	Comorbidity	3.760 (-23.647;31.167)	0.788	22.976 (-0.838; 46.790)	0.059
Occupation	Unemployed	-9.003 (-33.245; 15.245)	0.457	5.475(-14.465; 25.416)	0.590
	Farmer	-24.773 (-50.437; 0.892)	0.059	12.585 (-8.773;33.943)	0.248
	Worker	-67.096 (-97.387; -36.806)	0.000	-65.020 (-90.202; -39.838)	0.000
	Others	1		1	
Psychiatric classification	Schizophrenia	74.396 (31.533; 117.259)	0.001	31.930 (-3.548; 67.408)	0.078
	Bipolar disorder	24.177 (-20.925; 69.278)	0.293	19.101 (-17.901; 56.103)	0.312
	Other types	1		1	
Marital status	Single	-26.387 (-55.412; 2.638)	0.075	-4.381 (-29.126; 20.363)	0.729
	Married	-2.616 (-30.917; 25.685)	0.856	-8.678 (-31.990; 14.634)	0.466
	Divorced	1		1	

## Discussion

### Application prospects and therapeutic drug monitoring of clozapine

With the increasing awareness of the need for personalized treatment, therapeutic drug monitoring (TDM), which combines quantitative blood drug concentrations, pharmacological interpretation, and therapeutic guidance, has been introduced into drug therapy as a precision medicine tool [15]. TDM allows for individualized pharmacokinetic therapy by taking into account individual differences in pharmacokinetics [11]. It has become routine practice to quantify the plasma concentration of a large number of neuro psychotropic drugs, especially first-generation and second-generation antipsychotics and mood stabilizers [16]. At the same daily dose, as patients differ in their ability to absorb, distribute, metabolize and excrete drugs due to concurrent disease, age, concomitant medication or genetic abnormalities, the homeostasis concentration of a drug in vivo can vary more than 20-fold between individuals [17–23]. This study found a correlation of 0.594 between the daily dose and serum concentration of clozapine, which was a moderate correlation level (0.5≤|r|<0.8), and this result is similar to the published study [24], in which *r* = 0.49 (*p* < 0.001). This reflects that the metabolism of clozapine varies greatly among individuals. Therefore, in the clinical application of clozapine, individualized medication and drug concentration monitoring become necessary and more meaningful.

### Discussion of clozapine dose, concentration, and C/D values

Clozapine is mainly used for the treatment of treatment-resistant schizophrenia and schizoaffective disorder [25]; in addition, clozapine has also been used in the off-label treatment of bipolar disorder [3, 26], major depressive disorder (MDD) [27], and Parkinson’s disease (PD)[25, 28, 29]. The indications for clozapine in the drug label approved by China include that it may be effective for some patients who have failed or are not effective with traditional antipsychotics. It is also used for the treatment of agitation and hallucinatory delusions of mania or other psychotic disorders. The unique pharmacological properties and curative effects of clozapine make it gradually applied by psychiatrists beyond the drug label. In the study, clozapine was used by 75.92% of patients with schizophrenia, 22.25% of patients with affective (mood) disorders, and 1.83% of patients with other mental disorders. This indicates that clozapine is used in off-label treatment in the study area and suggests that clozapine has greater potential application value. Due to the side effects of clozapine, clozapine remains an underprescribed medication [28], and caution should be taken when using clozapine for off-label treatment.

Following the instructions for clozapine (Shandong Renhetang Pharmaceutical Co. Ltd), the treatment dose of clozapine was 201–400 mg/day, and the maintenance dose was 100–200 mg/day. The average dose of clozapine in the study population was 191.02 ± 113.47 mg/day, and doses of 50–350 mg/d accounted for 87.50% of the population, which means that 50–350 mg/day may be the effective therapeutic dose for the population in the study region. This result is different from the recommended dose of 300–600 mg/day but is similar to the study conclusion of Jose et al. Jose’s study showed that the average clozapine dose in Asian countries was less than 300 mg/day [30]. More research is needed to determine whether there are regional or ethnic differences in the therapeutic dose of clozapine.

The average serum concentration of clozapine in this study was 326.15 ± 235.66 ng/mL, lower than the Arbeitsgemeinschaft für Neuropsychopharmakologie und Pharmakopsychiatrie (AGNP) recommended effective and safe concentration of 350–600 ng/mL [31, 32]. The population within the concentration range of 350–600 ng/mL only accounted for 28.07% of the population in this study, while the population with < 350 ng/mL concentration accounted for 62.77%. The serum concentration range of 50–600 ng/mL accounted for 86.20% of the subjects in this study; therefore, the treatment concentration of clozapine recommended in this study area may be more appropriate at 50–600 ng/mL. The therapeutic reference scope of clozapine in treatment-resistant schizophrenia is still controversial [33], and some existing studies suggest keeping clozapine levels above 350 ng/mL before considering dosing [34], which may not be appropriate for all populations.

There were significant individual differences in the C/D ratio, which are affected by genetic, personal, and environmental factors [35, 36]. The C/D ratio indicates the clearance rate of drugs [37]; the smaller the C/D ratio is, the faster the drug clearance/metabolism is. In this study, the psychiatric patients were grouped according to the daily dose, the serum drug concentration increased and the C/D ratio decreased as the daily dose increased. The larger the daily dose was, the smaller the C/D ratio, indicating that the larger the dose was, the faster the clearance. Therefore, it is more important to carry out drug concentration monitoring in people taking large doses than in people taking small doses to ensure effective doses. In this study, the average C/D ratio was 1.94 ± 1.25, which was higher than the 1.57 in the Asian population and 1.07 in Caucasians in the study results of Jose et al. [30]. The results indicate that the metabolic rate of clozapine in the population of the study area might be slower and that a lower daily dose may be required to achieve an effective serum concentration. In this study, both the serum concentration and C/D ratio of females were higher than those of males, which was similar to the results of scholars such as Castberg [38], Michaelaet [39] and Jönsson [40] and consistent with the drug instructions. Similarly, the metabolic rate of clozapine was higher in males than in females.

### Discussion of influencing factors

In this study, the results showed that the older the age, the lower the required clozapine dose and the slower the metabolism, which suggests that serum drug concentration needs to be monitored among older individuals to prevent drug-related side effects caused by high serum concentration. The 10–17 year age group had the highest clozapine dose, the smallest C/D value and the smallest serum concentration. This may be because the metabolic rate of clozapine was faster in the 10–17 year age group, so a slightly higher dose was required to reach the serum effective therapeutic concentration. Our follow-up study focused on the association between different ages and the concentration of effective psychiatric medications.

Compared to outpatients, inpatients have similar daily doses, higher serum concentrations and slower metabolism rates. This may mean that there should be different standards for serum concentrations in inpatients and outpatients.

The daily dose of clozapine did not differ among patients with different occupations, but unemployed patients had higher serum concentrations and slower metabolic rates. This may be because unemployed patients have less physical labor than employed patients, and physical activity increases blood circulation and drug metabolism, so unemployed people have the highest C/D ratio or lower metabolic rate. This suggests that occupation may be one of the factors that predict serum clozapine concentration, and psychiatrists should consider occupation factors when prescribing dosage of clozapine. Further studies are needed to explain the association between occupation and clozapine metabolism.

Therefore, the monitoring of serum concentration should be strengthened in unemployed patients. Compared with psychiatric patients, patients with comorbidities have a smaller dose and slower metabolism; therefore, concentration monitoring in patients with comorbidities should be strengthened.

### Limitations of this study

The major enzymes and efflux transporters involved in the metabolism and distribution of clozapine include CYP1A2, CYP2C19, CYP3A4, and P-gp (ABCB1) [21, 41–43]. Some drugs, such as carbamazepine, phenobarbital, phenytoin and rifampicin, can induce CYP1A2, CYP2C19, CYP3A4, P-gp (ABCB1) and other enzymes or ABC transporters [44–46]. The combination of these drugs with clozapine may accelerate the metabolism and distribution of clozapine. Such drugs were not included in this study and should be included in subsequent studies. Some drugs, such as fluvoxamine, enoxacin and phenylpropanolamine, fluoxetine and norfluoxetine, omeprazole, macrolobemide, and voriconazol, can inhibit the activity of the CYP1A2 enzyme and CYP2C19 enzyme [47–51]. These drugs can inhibit the metabolism of clozapine and increase the blood concentration of clozapine. These drugs were not included in this study, and the interaction between these drugs and clozapine could not be analyzed, and subsequent studies should be optimized. Future studies should include drug interactions and clozapine-related metabolic genes.

The results of a previous study suggest that smoking is inversely associated with clozapine blood levels or decreases clozapine blood levels [39]. One mechanism that has been reported is that smoking can induce the CYP1A2 enzyme and accelerate the metabolism of clozapine [52]. Therefore, smoking patients should increase the dose of clozapine appropriately according to clinical needs and conduct blood concentration monitoring. Future clozapine-related studies should assess smoking.

## Conclusions

In summary, the metabolism of clozapine in psychiatric patients is affected by a variety of factors and varies greatly among individuals. It is important to monitor the drug concentration when taking clozapine to determine the optimal therapeutic concentration range to guide treatment and achieve accurate treatment [17].

## Data Availability

Data and material are available from the corresponding author upon reasonable request.

## Abbreviations

C/D: concentration/ dose
TDM: Therapeutic drug monitoring
Cmin: steady state valley concentration
HPLC: high-performance liquid chromatography
MDD: major depressive disorder
PD: Parkinson’s disease
